# Cleaning Teeth

**Published:** 1880-02

**Authors:** G. A. Mills


### ARTICLE IV.

### *Cleaning Teeth.*

BY G. A. MILLS.

(Read before the First District Dental Society.)

How many people who call upon the dentist for his services understand the full import of the heading of this article? How many dentists have anything like a fair degree of understanding regarding this matter? How many dentists make it the first object in their examination of the mouth? I think I can suggest a few directions that would come under the head of "practical hints to the dentist," and if accepted and followed, the result will bring so much satisfaction both to the patient and the operator as to permit of no future departure from the practice. I hear the remark all along the line, Who does not clean teeth? Echo answers, "Who does?" A few do; but close observation proves very conclusively that there is far from being anything like a respectable number who do set to work with the same degree of zeal that is manifested in the other departments of practice. Patients come to us to have their teeth examined, and the common practice is to look for decayed teeth. I say the *common* practice. Now, I do not think this is altogether so, because of ignorance or wickedness; and yet, to some extent, it may be a little of both. As dentists, we are morally bound to preach the gospel of saving the teeth, and also to be zealous for their *healthy surroundings;* for what does it profit the patient to have the teeth filled if the diseased conditions of the contiguous parts are sweeping them to destruction by destroying both the soft tissue of the gum and the hard of the sockets. "Cleaning teeth" has meant, heretofore, too much improving the facial appearance of a few front teeth—(A person going to a party or soon to be married, calls to get a fifty cent shine.) The operation of cleaning teeth means, according to the present-day standard, removing all debris from the cutting edge to apex of the root (if it be exposed,) all corroded surfaces, all stains, &c., and to wind up with a final polish, particularly on the enamel surface. As a practice, this should be the first step, and then every intelligent observer can learn the real condition of the teeth. This operation is verily the true method of examination. Now, to make this effectual and appreciated, patients calling to have their teeth examined should be informed that an appointment must be made which, as a rule, they will readily agree to. At the appointed time commence and clean each tooth from A to Z. If there be deposits of salivary calculus remove them (I would say a-la-Riggs) by any method by which thoroughness can be secured. This being done, by the use of corrundum disks (to a large extent,) stains, depressions, defects of tooth structure, &c., can be removed. For giving a smooth surface, Arkansas stone disks, with the free use of water, are very efficient. A very effective method for polishing about the neck and other surfaces of the teeth, is to place a fissure bur in the engine and wind about it a piece of cotton, so making readily an elastic cone. Another valuable method is the use of the string and tape polish ( which I described in my series of articles on the so-called Rigg's Disease, published in *Cosmos*, 1877,) to be used with moistened chalk and tooth powder. The final polishing should be done with the brush-wheel and the engine, using the polishing materials above referred to. Now, after this has been performed as thoroughly as it ought to be, and the beneficial result demonstrated, I think this method will impress the operator as of so much value that he will not hesitate to make it the rule of his practice ever thereafter; my word for it, as well as the testimony of those who practice it, no service performed in our offices will bring forth more evidence of real appreciation than this. A service of the nature I have described will, in ordinary cases, require at least one hour's time, but many cases will need much longer treatment. We will see, now, that an hour or more of the office time has been consumed and acknowledged by the patient to have been well spent. I think we have that which will militate against the remark so often heard in our societies and from those who ought not to make it, viz.: "That the time spent in such operations is all very well, but the patients will not pay for it." This is not true; they will pay, and liberally too. This matter of cleaning teeth should be well considered by young practitioners, for they can in no way pave so ready and so sure a path to a successful practice as by starting in this way. This will be an easy thing for a young practitioner to do. Alas! how many a one pines behind the diploma hanging on the wall of his primitive office, because of watching and waiting for patients who so long delay their coming. I predict that he who adopts as his motto' "clean teeth," need have no fear for the full employment of his time. This service well understood, will give the practitioner greater success in his professional life than all theories of the so-called "New Departure" system could. A dentist deficient in all the departments of practice, and zealous in the cleanliness of teeth, will be more successful in saving them, than one more efficient in general practice and neglectful in this particular. Look through all the discussions of our societies, and how much is said upon this subject? Cleaning teeth includes also securing a healthy mouth, and it is the most practical question of the hour, which *must* and *will* in time be in advance of all others. He who holds the position of teacher in the schools should spend more time in impressing the importance of this practice on the minds of his pupils than is at present the case. I have visited some of the schools and have quizzed the students on this subject, and found that it was receiving but a passing word in the teachings. But I need not have quizzed to find that out; silent but undeniable witnesses told the *true story* as I passed from chair to chair in the clinical department. Not in a single instance did I find this matter receiving any attention. I would suggest (as the importance of this subject bears so fully on me at this time,) that some student in the institution should have a clinic on his own teeth *first*, by some one who has the matter at heart and understands the full import of it, and then have the student operate, *each* on the *other*, and by this means an impression can be made which will transcend even an earnest effort in the lecture-room.

Up and down the avenues of our professional life we hear the declaration of those who have tarried long at the chair, that "Dentistry is a failure," "Sooner or later the forceps must come and put an end to the life effort." "Dentists do more harm than good," "Incompatibility of filling material," "Defective operative ability," "Cohesive gold," &c. Now let every one ask himself, "Have I given the attention to this subject which it deserves?" "Have I fully demonstrated in my practice the value of it?" Only a few days since one of our most brilliant fillers acknowledged that he had not given his attention to cleaning the teeth as fully as he felt he ought to have done. We must reverse the order of our clinics if we will make them most successful, and make this service occupy the first place in the clinic room—we will thus give it a potentiality that will be felt and be productive of great good. Until this is done, we make an almost useless expenditure of time and mental energy in trying to discover that which will put us in the way of solving the problem of saving the teeth, and will answer the question, "Why do teeth decay?" Of course, I do not mean to be understood that this practice alone settles the etiology of dental caries. As a truism—defective tooth structure admits of the possibility of decay, for the typic tooth will resist it. All unclean and unhealthy mouths are in a condition to accelerate dissolution of the soft as well as the hard tissues, and the result in the non-resisting constitution is general debility from a lesser to a greater degree. It is not at all uncommon to be able to trace directly to the unhealthy condition of the mouth and teeth local diseases of the eyes, ears, nasal cavities, and vocal organs; while in not a few cases the mucous membrane is deranged from the lips throughout the entire track of the alimentary system. The more attention I give to this subject, the more I am confirmed in the belief that we have as yet but a faint conception of the deleterious effects of a diseased oral cavity upon the general health. We cannot be too zealous in following out the line of practice which I have recommended, for by it to us is committed the responsibility of keeping a healthy vestibule of this temple of the human body. I predict the time is not far distant when the general physician will be forced to take cognizance of this fact, and will acknowledge the results of an intelligent practice by us in this field, to a far greater extent than he now does. We have come to understand this, that *all* inflammatory action is chemical. In its incipiency it may be circumscribed, and, in proportion as constitutional resistance is present, it is held in check until an additional complication is coupled with the first invasion, when the inflammatory action becomes intensified. Nature s now called upon to do extra duty, and the rallying of ithe vital forces to the point of attack often reveals an unknown weakness in some other part of the system, which develops to such an extent as to produce general debility. —*Dental Miscellany.*

